# Outwelling of nutrients into the Pasur River estuary from the Sundarbans mangrove creeks

**DOI:** 10.1016/j.heliyon.2022.e12270

**Published:** 2022-12-12

**Authors:** Jahid Hasan, Dinesh Chandra Shaha, Sampa Rani kundu, Minhaz Ahmed, Shahroz Mahean Haque, Farhana Haque, Md. Emranul Ahsan, Salman Ahmed, Md. Iqbal Hossain, Mohammad Abdus Salam

**Affiliations:** a Coastal and Marine Dynamics Laboratory, Department of Fisheries Management, Bangabandhu Sheikh Mujibur Rahman Agricultural University, Gazipur 1706, Bangladesh; b National Oceanographic and Maritime Institute, 10/8 Eastern Plaza, Sonargaon Road, Hatirpool, Dhaka 1219, Bangladesh; c Department of Agroforestry and Environment, Bangabandhu Sheikh Mujibur Rahman Agricultural University, Gazipur 1706, Bangladesh; d Department of Fisheries Management, Bangladesh Agricultural University, Mymensingh 2202, Bangladesh; e Department of Genetics and Fish Breeding, Bangabandhu Sheikh Mujibur Rahman Agricultural University, Gazipur 1706, Bangladesh

**Keywords:** Estuary, Creeks, Coastal region, Fishery resources, Chlorophyll-a

## Abstract

The Pasur River estuary (PRE), the largest estuary in the Sundarbans mangrove area, provides vital fishery resources and supports millions of livelihoods in the southwestern coastal region of Bangladesh. This study focused on the tidal and run-off effects on the outwelling of nutrients from the Sundarbans mangrove creeks to the PRE. Spatial and temporal variations of nutrient and chlorophyll-a concentrations were assessed by water sampling at 11 stations in the study area from January to December 2019. Dissolved inorganic nutrients and chlorophyll-a were analyzed by standard methods using a spectrophotometer. In the tidal mangrove creeks, dissolved inorganic nitrogen, phosphate, and silica concentrations were significantly higher (*p < 0.05*) during the spring tide than those during the neap tide, suggesting that these nutrients were flushed from the mangrove area by the inundation and tidal mixing of the spring tide. In general, chlorophyll-a (mean ± SD) concentrations in the PRE and the tidal mangrove creeks were 5.62 ± 1.30 μg/L and 9.03 ± 0.59 μg/L in the wet season, respectively. During the dry season, the chlorophyll-a decreased to 4.37 μg/L ± 0.68 and 4.94 ± 1.52 μg/L in the PRE and the tidal mangrove creek, respectively. The amount of nutrients outwelled from the mangrove creeks to the estuary was 1.53 ± 0.67 mg/L DIP, 0.001 ± 0.0004 mg/L DIN, and 1.38 ± 0.48 mg/L dissolved silica. DIP, silica, and chlorophyll-a concentrations were significantly higher (*p <* 0.05) during the spring tide compared to the neap tide, but salinity was not significantly (*p >* 0.05) different between the two tidal levels. This study showed that the mangrove creeks formed an important link in transporting nutrients from the mangrove forest to the estuary.

## 1 Introduction

The estuary is a semi-enclosed coastal water feature having free access to the open sea. It is in this transitional area that fresh water from land drainage is combined with sea water and creates the brackish water condition (Cameron and Pritchard, 1963; Calegario et al., 2015; Hasan et al., 2022). Due to their diverse microhabitats that range from freshwater to marine conditions, tidal mangrove creeks and estuaries are exceptionally active and productive ecosystems with great biodiversity (da Costa et al., 2011; Fernandes et al., 2018). However, estuaries only make up about 10% of the ocean's surface, while being crucial to the world's biogeochemical cycles, which include the carbon, nutrient, and nitrogen cycles (Lisitsyn, 1995; Gebhardt et al., 2005; Bernardino et al., 2022). Estuarine ecology is significantly influenced by seasonal variations, physicochemical traits (temperature, salinity, and nutrients), and hydrological factors like rainfall and tides (Valenzuela et al. 2021).

The Pasur River estuary (PRE), which is situated in the southwest coastal region of Bangladesh, is the largest and most prominent river in the Sundarbans mangrove environment (Islam, 2011). The Sundarbans Forest, which is situated between land and water on tropical and subtropical coasts, is the largest continuously productive mangrove wetland ecosystem in the world (Kathiresan and Bingham, 2001; Rahaman et al., 2013; Kathiresan and Bingham, 2001). India makes up the remaining 38% of the overall land, with the Khulna region in southwest Bangladesh making up the remaining 62% Siddiqi (2002). The Sundarbans in Bangladesh are made up of 1,874 km^2^ of rivers, streams, creeks, and canals and 6,017 km^2^ of mangrove forests, wildlife sanctuaries, and sand bars (Wahid, 1995; Siddiqi, 2001). By increasing production and preserving a wide variety of biodiversity, mangroves have increased the value of other coastal and marine resources, such as coastal and marine fisheries. Huge amounts of organic matter and nutrients may be exported by mangrove estuarine habitats to coastal marine environments, which has a significant effect on the biological productivity of the estuary (Pamplona et al., 2013; Magalhaes et al., 2015; Passos et al., 2022).

According to a hypothesized process called "outwelling," surplus carbon produced annually by coastal salt marshes and mangroves is "outwelled" as organic nutrients and debris into the surrounding ocean or coastal environment, increasing the productivity of neighboring fisheries or other coastal plants. Additionally, outwelling nourishes plankton colonies and promotes activity (Wolanski, 2007). However, tidal and climatic circumstances frequently affect the distribution and behavior of nutrients. During the wet season, river overflow floods the mangrove swamp (in the case of riverine forest type), trapping land-derived material that is later outwelled by tidal mixing and flows by inundation. Outwelling transports inorganic nutrients, dissolved organic matter, and particulate organic matter from the mangrove creeks to the estuary during each tide cycle (Ridd et al., 1988; Dittmar, 1999; Passos et al., 2021). According to Odum (2002), outwelling is not always a constant phenomenon that is altered by heavy rains or flash floods (the larger the inundation, the greater the outwelling). It is also impacted by the geomorphology of the estuary and the amplitude of the tides. Because outwelling is inversely correlated with an estuary's primary output, salt marshes with high productivity produce more outwelling. In estuaries surrounded by significant coastal wetlands, it is more obvious and frequent. Pulses of outwelling occur in response to flooding, precipitation, productivity, and tidal fluctuations. In certain cases, macrofauna and algae are pushed into the water column instead of fertilizers, but the impact is similar in terms of luring little fish and sustaining the productivity of the marine ecosystem. However, tidal and weather factors frequently affect the distribution and behavior of nutrients. Inorganic nitrogen concentrations in a mangrove creek rose after heavy rains or when tides flooded the forest base (Thong et al., 1993). Water quality is determined by its physical, chemical, and biological characteristics, and each of these variables has an effect on aquatic creatures' survival and reproduction, either directly or indirectly (Boyd and Tucker, 1998; Balqis et al., 2019; Hilaluddin et al., 2020). As a result, the productivity of the water body is directly related to the availability of nutrients. In order to maintain a healthy aquatic environment and promote the production of aquatic animals like fish without making the ecosystem eutrophic, nutrient concentrations must be within an acceptable range.

Information on nutrient dynamics is mostly limited to rivers and estuaries in the Bangladesh Sundarbans (Rahaman et al., 2013; Rahaman et al. 2014; Zinat et al., 2021; Kamruzzaman et al., 2019). Worldwide, information is also limited, especially with regards to nutrient changes during spring and neap tide (Tanaka and Choo, 2000), whereas research findings on nutrient dynamics from tidal mangrove creeks are very rare (Nion et al., 2020; Jahid et al., 2022). Despite the enormous potential of this mangrove forest, none of the previous investigations focused on the outwelling of inorganic nutrients from mangrove creeks to the Pasur River estuary. Thus, the current study was undertaken to investigate the outwelling of inorganic nutrients and dissolved organic matter from the tidal mangrove creeks to the Pasur River estuary.

## 2 Materials and methods

### 2.1 Study area

**Figure 1:**
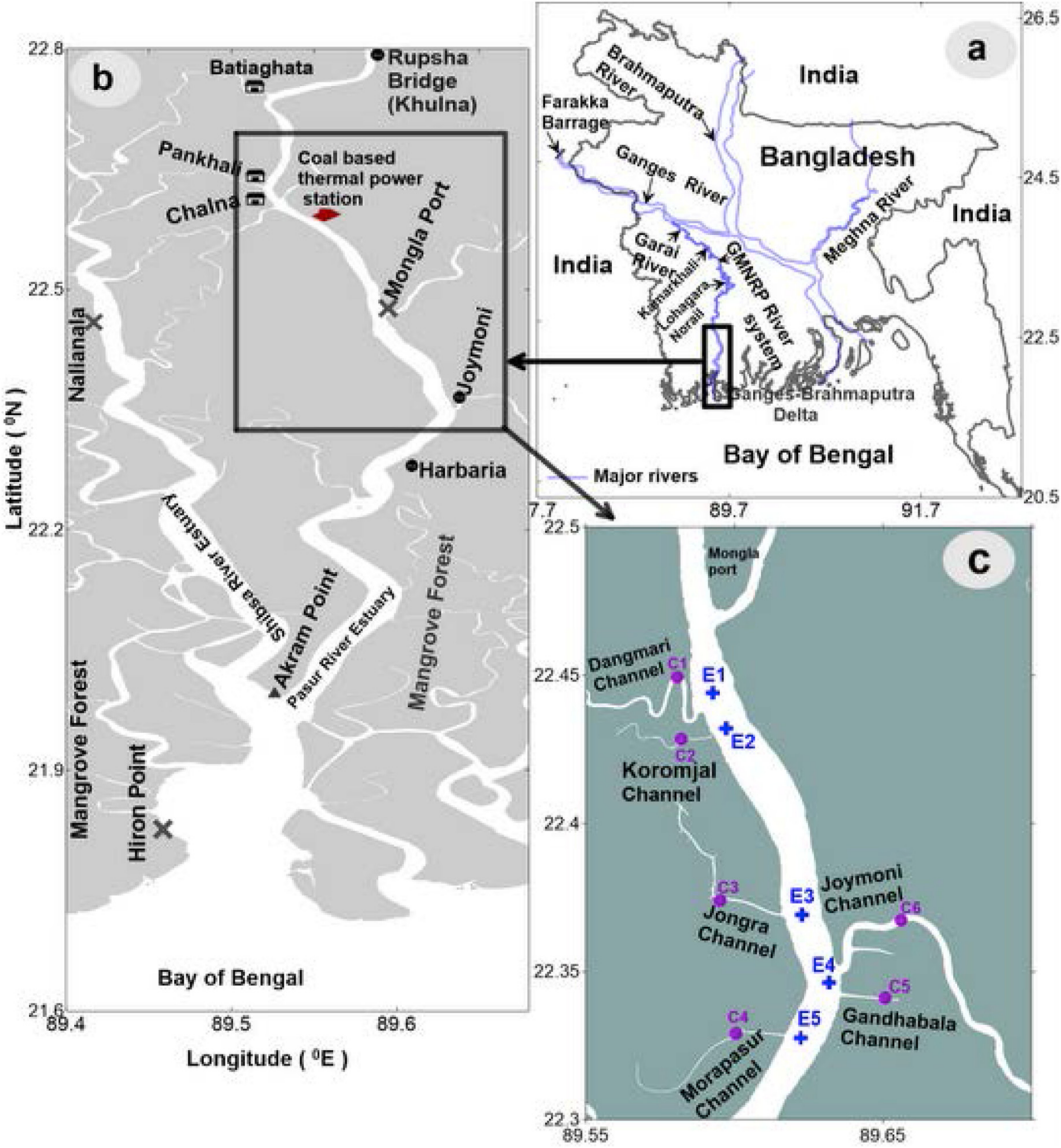
Study area and sampling locations [Mangrove creeks (purple dots): C1 (Dangmari Channel), C2 (Koromjal Channel), C3 (Jongra Channel), C4 (Morapasur Channel), C5 (Gandhabala Channel) and C6 (Joymoni Channel); Pasur River estuary (blue cross): E1, E2, E3, E4 and E5]. (a) Bangladesh map, (b) Pasur River estuary, (c) Sampling sites.

The Gorai Madhumati Rupsha Pasur River System (GMRP), the primary supply of fresh water upstream to the Bay of Bengal, is located in the southwest coastal region of Bangladesh in the Sundarban (IUCN, 1997), the largest mangrove ecosystem in the world. The Pankhali, Chunkhuri, and Bhadra Channels connect the Pasur River estuary (PRE) to the Shibsa River estuary (SRE). Due to the nutrient enrichment from the outwelling from the nearby mangrove creeks, this ecosystem is thought to have high primary production and so provides excellent fishing grounds. The Sundarbans are located in the southwest of Bangladesh and West Bengal in India, covering an area of around 10,000 km^2^. Eleven sampling stations make up the study area (Figure 1a, b, c), six of which are found in tidal mangrove creeks (Dangmari channel: C1, Koromjal channel: C2, Jongra channel: C3, Morapasur channel: C4, Gandhabala channel: C5, and Joymoni channel: C6), and five of which are found in the main Pasur River (E1, E2, E3, E4 and E5). It took 21 km of the Pasur River to map the research region using Golden Software Surfer 11 (Golden Software Inc., CO, USA).

### 2.2 Sample collection

Water samples were taken from eleven sampling stations covering seven times between January and December 2019 to account for both the dry (January 2019, March 2019, April 2019, June 2019, and December 2019) and wet (August 2019 and October 2019) seasons. The Bangladesh Inland Water Transport Authority's (BIWTA) spring and neap tide forecasts were used to determine the sampling dates (Table 1). Geographic coordinates were calculated at each sampling site using a Garmin e-Trex GPS. In all eleven stations, surface water was sampled using a 1.5 L water sampler (Wildco-1520) at a depth of 0–5 m (euphotic layer), immediately filtered using Whatman GF/F (0.45 m) membrane filter paper with a vacuum pump, and then stored in the refrigerator in the dark until the laboratory analysis.

**Table 1:** Tidal data for the sampling times.

Year 2019							
**Date**	Jan. 29	Mar. 16	Apr. 25	Jun. 23	Aug. 24	Oct. 25	Dec.
**Tidal range (m)**	2.2 m	2.4 m	3.6 m	4.0 m	3.9 m	3.7 m	2.9 m
**Spring (S) or Neap (N) tide**	Neap	Neap	Spring	Spring	Spring	Spring	Neap
**Ebb (E) or Flood (F) tide**	E, F	E, F	E, F	E, F	E, F	E, F	E, F

A pH meter (sensION + EC71) and dissolve oxygen (DO) meter (HACH HQ30d) were used to measure the concentrations of pH and DO, respectively. The Coastal and Marine Dynamics Laboratory, Department of Fisheries Management conducted nutrient analysis, including estimation of nitrite, nitrate, ammonia, inorganic phosphate, and silicate (APHA, 1998; Hach Company., 2012). The data were obtained using a spectrophotometric method (HACH, DR-6000, Germany, S/N: 1824775). A conductivity-temperature-depth (CTD) profiler (Model: In-situ Aqua TROLL 200, In-situ Inc., Fort Collins, Colorado, USA) was used to take vertical salinity, total dissolved solids (TDS), and temperature profiles along the main axis of the Pasur River estuary and the adjacent mangrove creeks. Analyses of chlorophyll-a used the technique of (Parsons et al., 1984).

Using the "heatmaply" software, boxplot analysis was carried out as a complement (Galili et al., 2018). For each environmental variable, descriptive statistics (mean and standard deviation) were calculated. After the normality test (Shapiro-Wilk test) and homogeneity of variance test, a two-way ANOVA and Student's t-test for independent samples were conducted using SPSS 22.0 to detect whether and where significant spatial and temporal differences occurred among the study locations (Levene test). Principal component analysis (PCA) was used to analyze the correlations between environmental variables in the study area using R version 4.0.3 (Pinherio et al., 2020). The "FactoMineR" program was used to run the PCAs (Le et al., 2008; Galili et al., 2018).

## 3 Results

### 3.1 Nutrients

**Figure 2:**
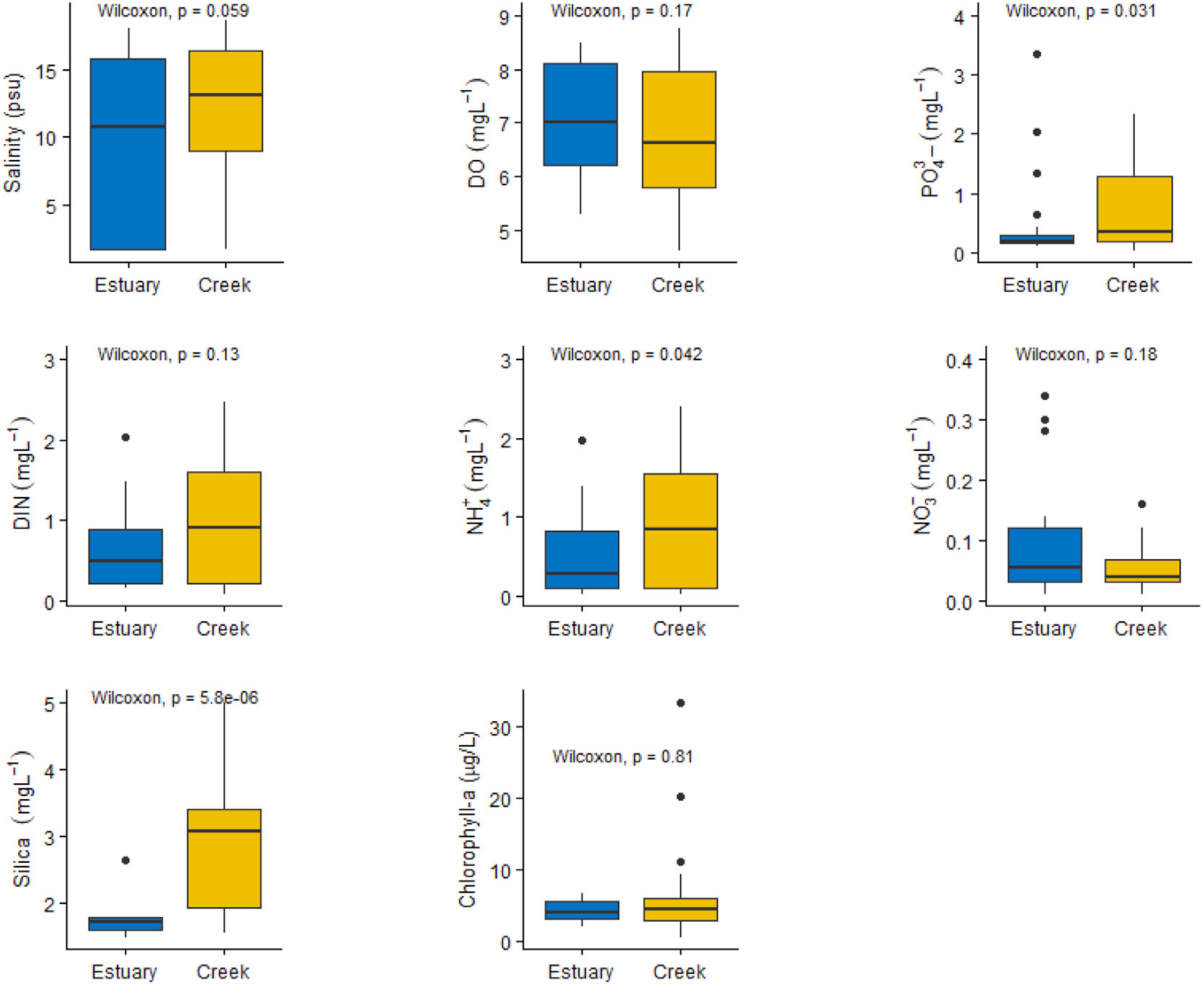
Spatial and temporal variation of major hydrochemical parameters (mean ± standard deviation) recorded at the survey stations during the dry season.

**Figure 3:**
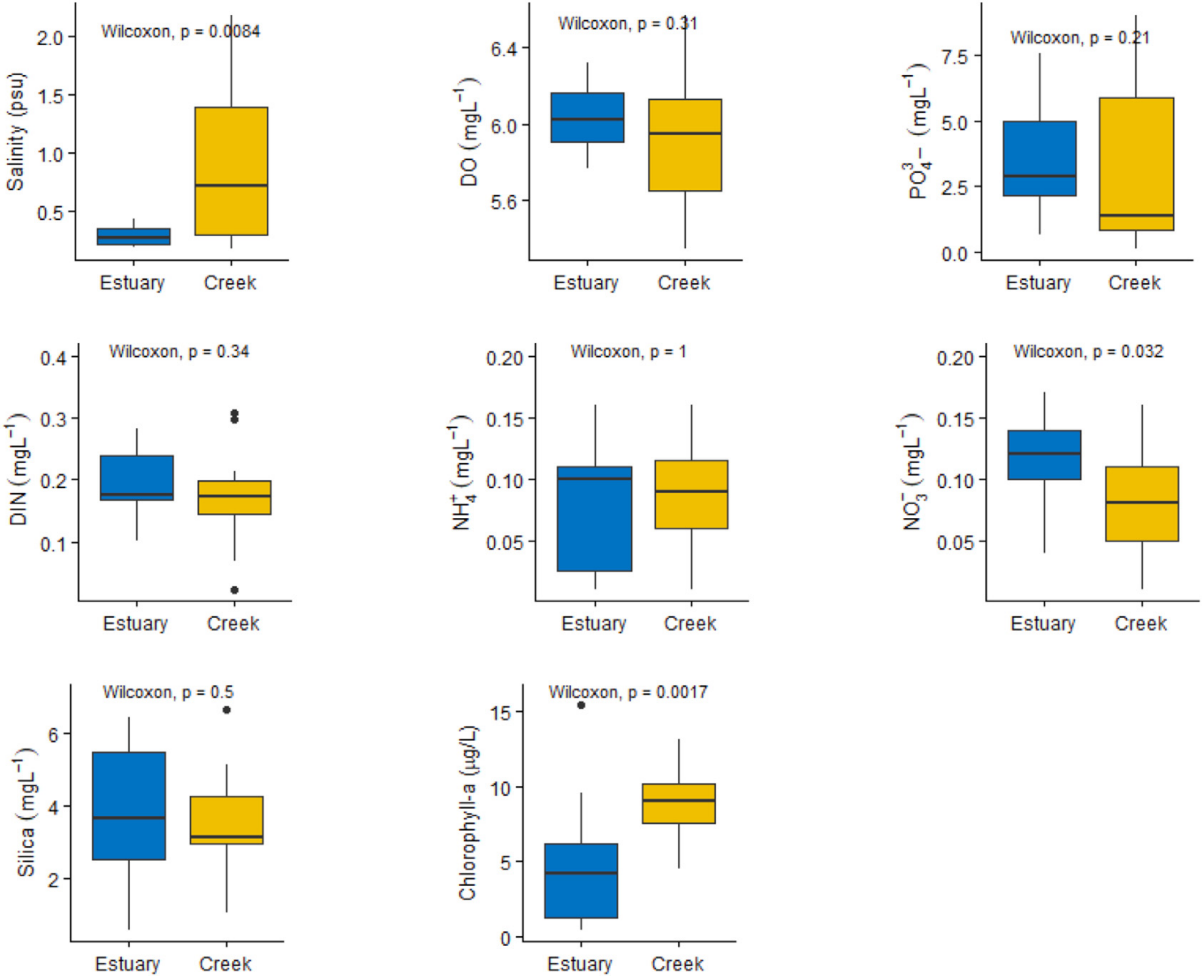
Spatial and temporal variation of major hydrochemical parameters (mean ± standard deviation) recorded at the survey stations during the wet season.

Dissolved inorganic nitrogen (DIN) at the study locations ranged from 0.07 to 2.32 mgL^_1^ with an average value of 0.65 ± 0.14 mgL^_1^, and their concentrations were significantly (*p* < 0.05) higher in the tidal mangrove creeks (0.71 ± 0.34 mgL^_1^) than those in the Pasur River estuary (PRE) (0.57 ± 0.25 mgL^_1^). Higher concentrations of DIN were found in the dry season (0.84 ± 0.55 mgL^_1^), whereas the wet season showed a lower DIN value of 0.18 ± 0.04 mgL^_1^ in the study area (Table 2 and Figures 2 and 3). In the dry season, DIN was higher in the tidal mangrove creeks (1.02 ± 0.63 mgL^_1^) than in the Pasur River estuary (0.62 ± 0.45 mgL^_1^). But, in the wet season, DIN was higher in the Pasur River estuary (0.20 ± 0.04 mgL^_1^) than in the tidal mangrove creeks (0.18 ± 0.04 mgL^_1^) (Table 2 and Figures 2 and 3).

**Table 2:** Mean, Standard deviation values of different ecological variables distribution in the tidal mangrove creeks of the Pasur River estuary.

ST	Season	Salinity (psu)	TDS (ppt)	pH	DO (mgL^_1^)	Chl-a (μg/L)	NO_3_ (mgL^_1^)	NO_2_ (mgL^_1^)	NH_4_ (mgL^_1^)	DIN (mgL^_1^)	DIP (mgL^_1^)	Silica (mgL^_1^)
*E1*	Dry season (non-monsoon)	11.570 ± 6.440	12.430 ± 6.670	07.750 ± 0.080	06.980 ± 0.610	03.990 ± 0.770	00.009 ± 0.004	00.048 ± 0.036	00.610 ± 0.520	00.670 ± 0.500	00.200 ± 0.050	02.650 ± 0.100
	Wet season (monsoon)	00.210 ± 0.030	00.380 ± 0.180	08.000 ± 0.0450	06.040 ± 0.060	03.650 ± 1.270	00.002 ± 0.001	00.160 ± 0.014	00.060 ± 0.040	00.220 ± 0.080	03.400 ± 1.550	05.430 ± 0.060
*E2*	Dry season (non-monsoon)	11.640 ± 6.510	12.360 ± 6.610	07.690 ± 0.700	06.880 ± 0.610	04.004 ± 0.690	00.005 ± 0.002	00.064 ± 0.040	00.810 ± 0.520	00.870 ± 0.710	00.220 ± 0.120	01.480 ± 0.067
	Wet season (monsoon)	00.270 ± 0.120	00.360 ± 0.150	07.740 ± 0.200	05.950 ± 0.130	03.650 ± 1.130	00.006 ± 0.002	00.140 ± 0.001	00.060 ± 0.040	00.206 ± 0.060	03.250 ± 1.520	03.590 ± 0.080
*E3*	Dry season (non-monsoon)	11.420 ± 6.370	12.180 ± 6.510	07.620 ± 0.120	06.730 ± 0.580	04.004 ± 0.720	00.005 ± 0.002	00.100 ± 0.060	00.440 ± 0.300	00.540 ± 0.230	00.170 ± 0.040	01.780 ± 0.082
	Wet season (monsoon)	00.280 ± 0.030	00.420 ± 0.110	07.950 ± 0.080	06.050 ± 0.130	04.990 ± 1.230	00.004 ± 0.001	00.085 ± 0.063	00.085 ± 0.063	00.174 ± 0.001	04.650 ± 2.510	06.380 ± 0.077
*E4*	Dry season (non-monsoon)	10.940 ± 6.070	11.890 ± 6.190	07.690 ± 0.110	06.780 ± 0.450	04.640 ± 0.710	00.010 ± 0.001	00.070 ± 0.040	00.740 ± 0.490	00.820 ± 0.490	00.405 ± 0.204	01.590 ± 0.040
	Wet season (monsoon)	00.280 ± 0.100	00.380 ± 0.150	07.840 ± 0.140	05.970 ± 0.130	08.120 ± 2.150	00.005 ± 0.001	00.090 ± 0.035	00.100 ± 0.001	00.200 ± 00.036	04.600 ± 3.164	01.860 ± 1.930
*E5*	Dry season (non-monsoon)	10.830 ± 5.780	11.950 ± 6.050	07.660 ± 0.080	06.730 ± 0.50	04.130 ± 0.540	00.006 ± 0.003	00.050 ± 0.032	00.570 ± 0.369	00.630 ± 0.340	00.220 ± 0.160	01.710 ± 0.050
	Wet season (monsoon)	00.830 ± 0.520	00.460 ± 0.210	07.630 0 ± 0.260	06.130 ± 0.130	05.190 ± 0.730	00.003 ± 0.002	00.100 ± 0.001	00.050 ± 0.030	00.150 ± 0.070	01.390 ± 1.030	05.110 ± 1.630
*ST*	**Season**	Salinity (psu)	TDS (ppt)	pH	DO (mgL^_1^)	Chl-a (μg/L)	NO_3_ (mgL^_1^)	NO_2_ (mgL^_1^)	NH_4_ (mgL^_1^)	DIN (mgL^_1^)	DIP (mgL^_1^)	Silica (mgL^_1^)
*C1*	Dry season (non-monsoon)	011.560 ± 6.470	12.230 ± 6.470	07.740 ± 0.090	06.780 ± 0.600	03.510 ± 0.590	00.009 ± 0.003	00.050 ± 0.019	00.960 ± 0.560	01.030 ± 0.900	00.990 ± 0.740	05.010 ± 0.110
	Wet season (monsoon)	00.220 ± 0.050	00.280 ± 0.040	07.830 ± 0.540	06.120 ± 0.160	07.350 ± 0.005	00.023 ± 0.020	00.120 ± 0.020	00.060 ± 0.030	00.190 ± 0.050	02.740 ± 1.440	02.730 ± 2.360
*C2*	Dry season (non-monsoon)	12.520 ± 6.120	11.450 ± 5.517	07.630 ± 0.080	06.420 ± 0.690	05.080 ± 1.910	00.014 ± 0.008	00.050 ± 0.042	01.160 ± 0.910	01.230 ± 0.880	00.660 ± 0.570	01.550 ± 0.110
	Wet season (monsoon)	00.550 ± 0.480	00.610 ± 0.480	07.670 ± 0.150	05.970 ± 0.090	09.730 ± 0.590	00.004 ± 0.001	00.057 ± 0.030	00.060 ± 0.030	00.120 ± 0.060	04.760 ± 2.190	04.120 ± 1.400
*C3*	Dry season (non-monsoon)	11.730 ± 6.110	12.470 ± 6.150	07.610 ± 0.070	06.350 ± 0.560	04.980 ± 1.070	00.006 ± 0.003	00.0440 ± 0.020	01.050 ± 0.580	01.110 ± 0.540	00.640 ± 0.470	01.990 ± 0.100
	Wet season (monsoon)	00.970 ± 0.170	01.030 ± 0.520	07.520 ± 0.250	05.730 ± 0.080	07.570 ± 0.090	00.007 ± 0.001	00.080 ± 0.040	00.110 ± 0.020	00.190 ± 0.070	01.270 ± 0.450	03.590 ± 0.090
*C4*	Dry season (non-monsoon)	11.120 ± 5.280	11.680 ± 5.770	07.640 ± 0.070	06.700 ± 0.050	04.730 ± 1.370	00.004 ± 0.002	00.050 ± 0.020	00.510 ± 0.270	00.560 ± 0.240	00.550 ± 0.410	03.410 ± 0.110
	Wet season (monsoon)	01.360 ± 0.670	02.870 ± 0.810	07.730 ± 0.040	05.690 ± 0.190	09.040 ± 0.850	00.009 ± 0.007	00.050 ± 0.040	00.080 ± 0.001	00.150 ± 0.040	03.090 ± 2.780	03.330 ± 1.290
*C5*	Dry season (non-monsoon)	11.120 ± 6.100	11.850 ± 6.200	07.620 ± 0.120	07.180 ± 0.690	09.840 ± 1.710	00.006 ± 0.002	00.060 ± 0.030	00.860 ± 0.510	00.930 ± 0.690	00.690 ± 0.430	01.940 ± 0.100
	Wet season (monsoon)	00.540 ± 0.130	00.660 ± 0.130	07.640 ± 0.120	06.130 ± 0.140	09.070 ± 0.800	00.006 ± 0.003	00.090±−0.020	00.150 ± 0.001	00.190 ± 0.010	02.570 ± 2.290	04.780 ± 2.600
*C6*	Dry season (non-monsoon)	11.350 ± 6.170	11.950 ± 6.200	07.700 ± 0.090	06.950 ± 0.50	06.690 ± 2.520	00.010 ± 0.009	00.070 ± 0.050	00.730 ± 0.600	00.810 ± 0.580	00.470 ± 0.360	03.070 ± 0.110
	Wet season (monsoon)	01.280 ± 0.310	00.410 ± 0.100	07.750 ± 0.010	05.820 ± 0.220	11.410 ± 1.230	00.006 ± 0.004	00.080 ± 0.060	00.090 ± 0.050	00.190 ± 0.020	03.50 ± 3.100	03.04 ± 0.040

(ST: Station; TDS: Total Dissolved Solids; DO: Dissolved oxygen; Chl-a: Chlorophyll-a; NO_3_: Nitrate; NO_2_: Nitrite; NH_4_: Ammonia; DIN: Dissolved Inorganic Nitrogen; DIP: Dissolved Inorganic Phosphate).

Dissolved inorganic phosphate (DIP) at the study locations ranged from 0.06 to 8.44 mgL^_1^ with an average of 1.30 ± 1.16 mgL^_1^ in all the sampling months. The mean DIP concentration was insignificantly higher (*p* > 0.05) in the tidal mangrove creeks (1.35 ± 1.26 mgL^_1^) than in PRE (1.25 ± 1.03 mgL^_1^) in all the sampling months (Table 2). Significantly higher (*p* < 0.05) concentrations of DIP were found in wet season (8.44–0.16 mgL^_1^) with a mean value of 3.20 ± 2.00 mgL^_1^, whereas the dry season showed a lower dissolved inorganic phosphate concentration (2.10–0.06 mgL^_1^) with an average value of 0.48 ± 0.32mgL^_1^ in the sampling sites (Table 2 and Figures 2 and 3). In the dry season, DIP was higher in the tidal mangrove creeks (0.72 ± 0.49 mgL^_1^) than in the Pasur River estuary (0.48 ± 0.11 mgL^_1^), but in the wet season, DIP was higher in the Pasur River estuary (3.65 ± 1.95 mgL^_1^) than in the tidal mangrove creeks (2.94 ± 2.04 mgL^_1^) (Table 2 and Figures 2 and 3).

Dissolved silica showed significant differences (*p* < 0.05) between the Pasur River estuary and the tidal mangrove creeks both spatially and temporarily. The mean dissolved silica concentration was significantly higher (*p* < 0.05) in the tidal mangrove creeks (3.24 ± 0.70 mgL^_1^) than in the Pasur River estuary (2.84 ± 0.41 mgL^_1^) in all the sampling months. In the dry season, dissolved silica concentrations were significantly higher (*p* < 0.05) in the tidal mangrove creeks (2.84 ± 0.10 mgL^_1^) than those in the Pasur River estuary (1.64 ± 0.06 mgL^_1^) in all the sampling months (Table 2 and Figures 2 and 3). In contrast, in the wet season, dissolved silica concentrations were significantly higher (*p* < 0.05) in the Pasur River estuary (4.02 ± 0.75 mgL^_1^) than in the tidal mangrove creeks (3.66 ± 1.29 mg/L) (Table 2 and Figures 2 and 3).

### 3.2 Chlorophyll-a

In the study area, chlorophyll-a ranged from 0.42 to 33.26 μg/L with an average value of 5.68 ± 2.38 μg/L. There was no significant variation (*p* > 0.05) was observed in the chlorophyll-a concentration between the tidal mangrove creeks (6.20 ± 2.36 μg/L) and the Pasur River estuary (4.80 ± 2.26 μg/L) (Table 2). This trend was visible in all the sampling seasons. The mean chlorophyll-a concentrations in the dry season and wet season were 4.66 ± 1.14 μg/L and 7.32 ± 0.91 μg/L, respectively. The mean chlorophyll-a concentrations were significantly higher (*p* < 0.05) in the wet season than in the dry season (Figures 2 and 3). Chlorophyll-a was highly correlated with phosphate (R = 0.230, *p* < 0.05), with no significant correlation with salinity (Table 3).

**Table 3:** Results of Pearson correlation analysis (R) for the investigated physical and chemical variables during monthly observation in the Pasur River estuary and its connected mangrove creeks.

Variables	Tem	Salinity	DO	pH	NO_3_	NO_2_	NH_4_	DIP	Silica	TDS	DIN	Chl-a
**Tem**	1											
**Salinity**	0.360∗∗	1										
**DO**	−0.916∗∗	−0.273∗∗	1									
**pH**	−0.087	−0.255∗	0.070	1								
**NO**_**3**_	−0.355∗∗	−0.543∗∗	0.404∗∗	0.189	1							
**NO**_**2**_	−0.122	0.051	0.128	0.116	0.234∗	1						
**NH**_**4**_	0.477∗∗	0.817∗∗	−0.414∗∗	−0.187	−0.429∗∗	0.033	1					
**DIP**	0.118	−0.376∗∗	−0.226∗	−0.081	−0.002	−0.109	−0.262∗∗	1				
**Silica**	0.551∗	−0.518∗	−0.467∗	−0.335	−0.039	−0.354	−0.150	0.206	1			
**TDS**	0.343∗∗	0.999∗∗	−0.257∗∗	−0.252∗	−0.543∗∗	0.057	0.809∗∗	−0.386∗∗	−0.564∗	1		
**DIN**	0.455∗∗	0.796∗∗	−0.385∗∗	−0.172	−0.342∗∗	0.091	0.995∗∗	−0.275∗∗	−0.158	0.788∗∗	1	
**Chl-a**	−0.078	−0.189	0.019	0.013	−0.038	−0.042	−0.200∗	0.230∗	0.156	−0.189	−0.212∗	1

∗ *p < 0.05, ∗∗p < 0.01,* (Tem: Temperature; DO: Dissolved oxygen; NO_3_: Nitrate; NO_2_: Nitrite; NH_4_: Ammonia; TDS: Total Dissolved Solids; DIN: Dissolved Inorganic Nitrogen; DIP: Dissolved Inorganic Phosphate; Chl-a: Chlorophyll-a).

### 3.3 Salinity

**Figure 4:**
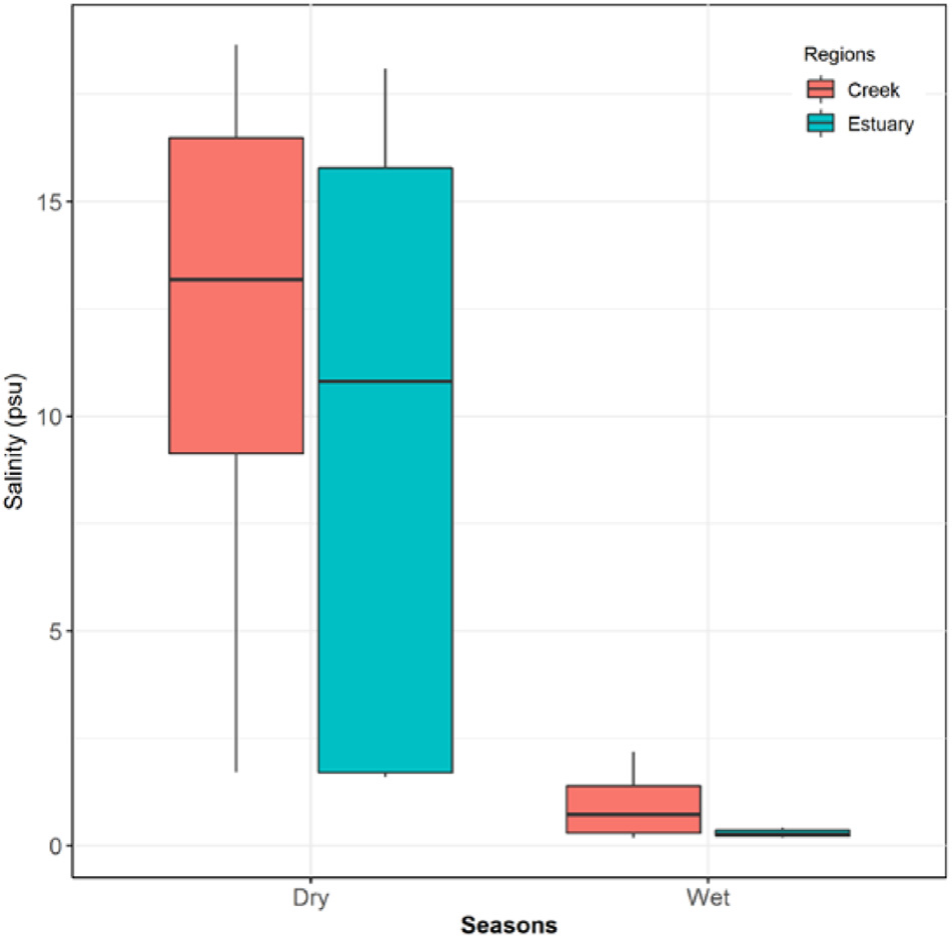
Salinity distribution in the Pasur River estuary and the tidal mangrove creeks (mean ± standard deviation) recorded at the survey stations during the study period.

Salinity ranged from 0.18 to 18.66 psu with an average of 8.34 ± 1.57 psu in the study area. Higher concentrations of salinity were found in the dry season (10.54 ± 3.05 psu), whereas the wet season showed a lower salinity (0.61 ± 0.11 psu) (Figure 4 and Table 2) in the PRE and the connected mangrove creeks. The salinity level in tidal mangrove creeks during spring and neap tides was 8.78 ± 6.61 psu and 8.12 ± 5.31 psu, respectively. Both the dry and wet season salinity values were higher in the tidal mangrove creeks (Dry: 11.56 ± 6.04 psu; Wet: 0.82 ± 0.30 psu) than in the Pasur River estuary (Dry: 11.28 ± 6.23 psu; Wet: 0.37 ± 0.16 psu) (Table 2 and Figures 2 and 3). Salinity steadily increased to a maximum during the dry winter season and then significantly dropped during the monsoon period.

### 3.4 Distribution of dissolved inorganic nutrients and chlorophyll-a during spring and neap tides

**Figure 5:**
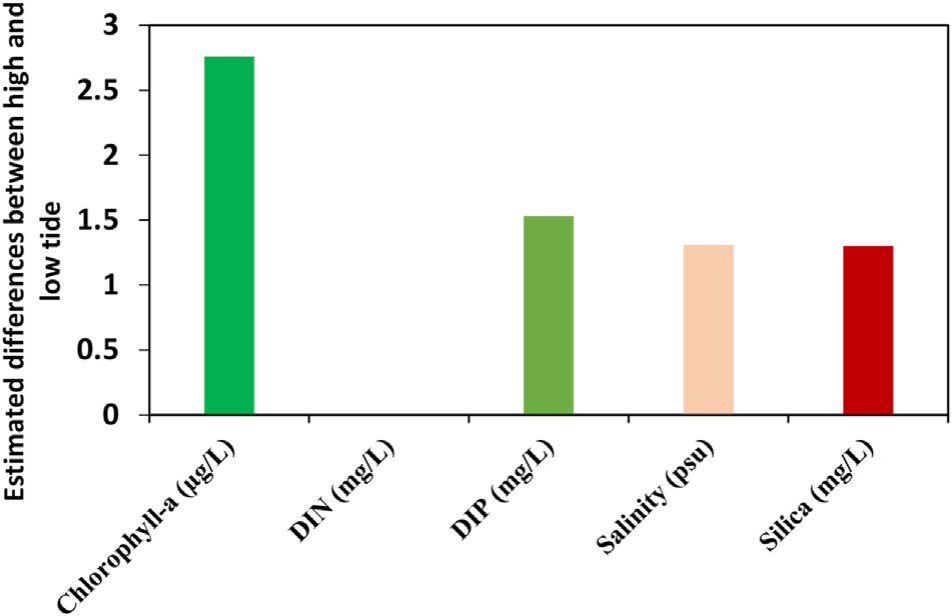
Amount of salt, nutrients and chlorophyll-a fluxes (outwelled) from the Sundarban mangrove creeks to the Pasur River estuary during the low tide.

Mean DIN concentration in the tidal mangrove creeks during the spring and neap tides was 0.87 ± 0.26 mgL^_1^ and 0.52 ± 0.17 mgL^_1^, respectively (Table 4). Similarly, mean DIP concentration in the tidal mangrove creeks during the spring and neap tides was 1.93 ± 0.43 mgL^_1^ and 0.53 ± 0.05 mgL^_1^, respectively. The mean silica value in tidal mangrove creeks during spring and neap tides was 4.11 ± 1.98 mgL^_1^ and 2.83 ± 1.14 mgL^_1^, respectively (Table 4). Salinity levels in tidal mangrove creeks during spring and neap tides were 8.78 ± 8.12 psu and 8.12 ± 5.31 psu, respectively (Table 4). Chlorophyll-a value in the tidal mangrove creeks during the spring and neap tide with a mean value of 6.85 ± 0.87 μg/L and 4.89 ± 0.85 μg/L, respectively (Table 4). Chlorophyll-a, DIP, pH and silica concentration varied significantly (*p* < 0.05) between the spring and neap tides. Similarly, ammonia, DIN, DO, pH, temperature and salinity varied significantly (*p* < 0.05) between the high and low tides. Nitrate and temperature varied significantly (*p* < 0.05) according to the interaction effects (spring∗ neap∗ high∗ low tides). In the wet season, chlorophyll-a, DIP, and silica concentrations were higher than in the non-monsoon season. The large differences in salinity and nutrients between inflowing (high water) and outflowing (low water) water in the mangrove creeks enable a reliable calculation of salt and nutrient export from the mangrove creeks (Figure 5). The amounts of salt and nutrients outwelled from the mangrove creeks to the estuary were 1.53 mg/L DIP, 0.001 mg/L DIN, 1.38 mg/L dissolved silica, 1.31 psu salinity, and 2.76 μg/L chlorophyll-a.

**Table 4:** Dissolved nutrients, salinity and chlorophyll-a distribution in the tidal mangrove creeks (spring vs neap; high vs low tides).

Station	Tide	Salinity (psu)	Chlorophyll-a (μg/L)	DIN (mgL^_1^)	DIP (mgL^_1^)	Silica (mgL^_1^)
*C1*	Low tide	11.56 ± 5.45	3.51 ± 1.27	1.02 ± 0.67	0.994 ± 0.5	3.68 ± 1.68
	High tide	1.70 ± 0.98	2.89 ± 0.79	0.21 ± 0.14	0.18 ± 0.04	2.48 ± 1.22
	Spring tide	8.72 ± 6.24	5.27 ± 2.55	1.15 ± 1.10	2.17 ± 1.6	2.73 ± 2.35
	Neap tide	7.79 ± 5.49	3.72 ± 1.24	0.26 ± 0.25	0.59 ± 0.38	5.01 ± 2.89
*C2*	Low tide	12.52 ± 7.56	5.09 ± 2.35	1.23 ± 0.76	0.67 ± 0.22	3.72 ± 1.67
	High tide	9.58 ± 3.23	4.285 ± 1.46	0.33 ± 0.13	0.24 ± 0.09	2.37 ± 1.11
	Spring tide	9.33 ± 8.12	7.55 ± 2.62	1.11 ± 0.8	2.79 ± 1.89	4.11 ± 1.40
	Neap tide	8.80 ± 4.77	4.91 ± 2.14	0.65 ± 0.54	0.57 ± 0.36	1.56 ± 0.67
*C3*	Low tide	12.39 ± 5.45	5.32 ± 2.79	1.12 ± 0.67	0.66 ± 0.32	3.84 ± 1.70
	High tide	9.18 ± 4.56	5.19 ± 2.34	0.44 ± 0.22	0.18 ± 0.08	2.35 ± 1.32
	Spring tide	9.06 ± 7.10	6.15 ± 2.46	0.85 ± 0.76	0.99 ± 0.61	5.05 ± 1.97
	Neap tide	8.13 ± 5.08	5.15 ± 2.01	0.84 ± 0.56	0.61 ± 0.38	1.99 ± 0.89
*C4*	Low tide	11.73 ± 5.69	5.08 ± 2.34	0.55 ± 0.23	0.47 ± 0.19	3.77 ± 1.64
	High tide	8.67 ± 3.45	4.56 ± 1.68	0.36 ± 0.17	0.14 ± 0.03	2.52 ± 1.09
	Spring tide	8.34 ± 6.66	7.41 ± 3.63	0.39 ± 0.27	1.91 ± 1.45	3.33 ± 1.29
	Neap tide	8.32 ± 5.13	4.04 ± 0.61	0.53 ± 0.33	0.45 ± 0.29	3.41 ± 1.45
*C5*	Low tide	11.12 ± 6.34	3.99 ± 1.35	0.93 ± 0.67	0.69 ± 0.31	3.66 ± 1.83
	High tide	1.67 ± 0.23	4.79 ± 2.01	0.16 ± 0.60	0.15 ± 0.04	1.90 ± 1.19
	Spring tide	8.37 ± 5.64	6.75 ± 4.28	1.01 ± 0.79	1.85 ± 1.18	4.79 ± 2.60
	Neap tide	7.74 ± 5.61	3.41 ± 1.63	0.34 ± 0.12	0.41 ± 0.25	1.94 ± 0.98
*C6*	Low tide	11.35 ± 6.90	6.69 ± 2.90	0.81 ± 0.57	0.47 ± 0.35	3.31 ± 1.55
	High tide	1.67 ± 0.47	4.79 ± 1.98	0.16 ± 0.07	0.15 ± 0.07	2.05 ± 0.98
	Spring tide	8.85 ± 5.89	7.96 ± 4.31	0.76 ± 0.65	1.89 ± 1.2	4.67 ± 2.26
	Neap tide	7.97 ± 5.78	8.15 ± 3.18	0.46 ± 0.35	0.6 ± 0.30	3.07 ± 1.56

### 3.5 Statistical analysis

**Figure 6:**
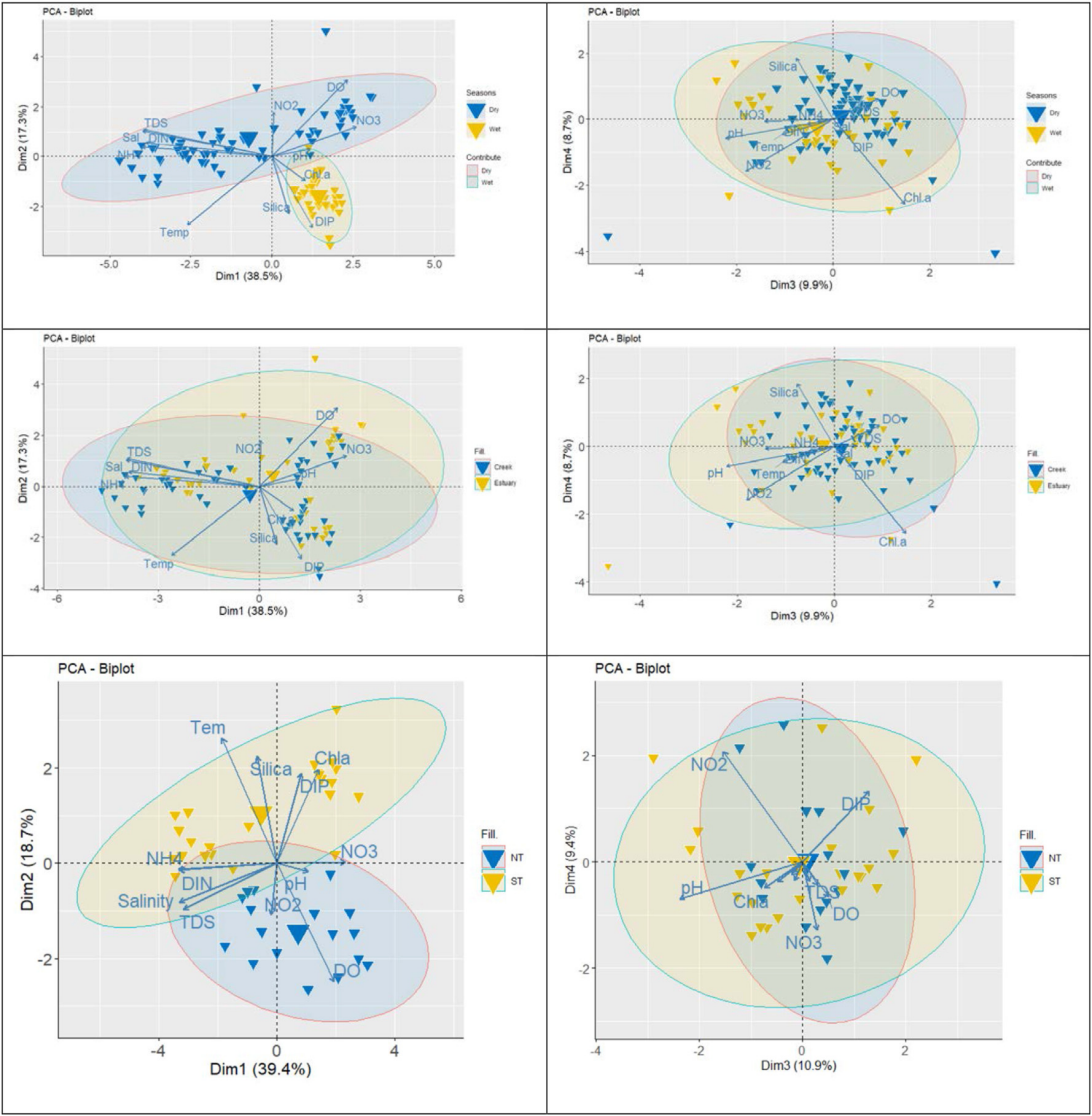
Graphical representation of the principal component analysis (PCA) of different ecological parameter distribution in the tidal mangrove creeks and Pasur River estuary (PRE) (Tem: Temperature; Chl-a: Chlorophyll-a; NO_3_: Nitrate; NO_2_: Nitrite; NH_4_: Ammonia; DIN: Dissolved inorganic nitrogen; DIP: Dissolved inorganic phosphate; ST: Spring tide and NT: Neap tide).

The multivariate analysis showed a seasonal gradient for the water quality parameters, forming two different groups for the dry and wet seasons as well as spring and neap tides (Figure 6). A strong negative correlation was observed between the spring and neap tides concentration of nutrients and environmental variables such as chlorophyll-a, silica, ammonia, DIN, DIP, water temperature, and salinity in the tidal mangrove creeks. The pearson correlations between the surface values of ecologically important variables are shown in (Table 3). Salinity showed a significant positive correlation with pH (R = −0.255, *p* < 0.05). Relevantly, it was negatively correlated with DIN (R = 0.796, *p* < 0.01), DO (R = −0.273, *p* < 0.01), DIP (R = −0.376, *p* < 0.01), and silica (R = −0.518, *p* < 0.05). Negative correlations between salinity and DO were highly significant (R = −0.273, *p* < 0.001) (Table 5).

**Table 5:** Results of Pearson correlation analysis (R) for the investigated physical and chemical variables during monthly observation in the Creeks of the Pasur River estuary.

Variables	Tem	Salinity	DO	pH	NO_3_	NO_2_	NH_4_	DIP	Silica	TDS	DIN	Chl-a
**Tem**	1											
**Salinity**	0.306∗	1										
**DO**	−0.883∗∗	−0.183	1									
**pH**	−0.136	−0.178	0.151	1								
**NO**_**3**_	−0.220	−0.554∗∗	0.326∗	0.125	1							
**NO**_**2**_	0.085	0.016	−0.071	0.200	−0.088	1						
**NH**_**4**_	0.509∗∗	0.850∗∗	−0.416∗∗	−0.150	−0.441∗∗	0.060	1					
**DIP**	−0.005	−0.363∗∗	−0.149	−0.271∗	−0.038	−0.063	−0.257∗	1				
**Silica**	0.587	−0.714∗	−0.382	−0.345	0.128	−0.383	0.358	0.358	1			
**TDS**	0.290∗	0.999∗∗	−0.167	−0.174	−0.561∗∗	0.016	0.841∗∗	−0.374∗∗	−0.765∗∗	1		
**DIN**	0.509∗∗	0.839∗∗	−0.408∗∗	−0.143	−0.397∗∗	0.072	0.999∗∗	−0.265∗	0.270	0.829∗∗	1	
**Chl-a**	−0.218	−0.328∗	0.158	0.203	0.036	−0.081	−0.318∗	0.169	0.595	−0.325∗	−0.324∗	1

∗ Correlation is significant at the 0.05 level, ∗∗ Correlation is significant at the 0.01 level, (Tem: Temperature; DO: Dissolved oxygen; NO_3_: Nitrate; NO_2_: Nitrite; NH_4_: Ammonia; TDS: Total Dissolved Solids; DIN: Dissolved Inorganic Nitrogen; DIP: Dissolved Inorganic Phosphate; Chl-a: Chlorophyll-a).

## 4 Discussion

Photosynthesis, growth, composition, and variety of plankton in an ecosystem are all impacted by changes in monsoonal patterns, which include variations in rainfall intensity, light, tidal changes, waves, and nutrient outwelling (Sodre et al., 2011; Magalhaes et al., 2015; Alongi, 2018). According to the phases of the moon, tides had a considerable impact on the concentrations of nutrients (Dittmar, 1999). Nutrient dynamics in mangroves and other wetlands as well as nutrient concentrations in mangrove creeks water can be greatly influenced by inundation regimes (Cohen et al., 2004). In comparison to neap tide, spring tide concentrations of DIN, DIP, salinity, silica, and chlorophyll-a were substantially higher. Similar trends have been identified for a few additional tropical estuaries around the coast of Brazil (Silva and Sampaio, 1998; Dittmar, 1999; Tanaka and Choo, 2000; Cohen et al., 2004; Cavalcanti et al., 2018). Other methods of nitrogen and phosphate retention used by mangroves include a vast pool of dead roots and tight immobilization of accessible solutes. Inorganic components including soil aluminum, iron, calcium, and sulfides are strongly bonded to phosphorus, and the majority of the dissolved nitrogen in soil is ammonium rather than nitrite and nitrate (Alongi, 2018; Thong et al., 1993). The DIN and DIP concentrations in the water column are largely attributed to the release of anoxic interstitial waters from the surface mangrove sediments by tidal wash-out, suggesting that these nutrients are flushed from the mangrove area by inundation and tidal mixing, similar to the Brazilian coast. During the dry season, the average DIN and DIP content were as higher in the tidal mangrove creeks than in the PRE (Santos et al., 2008). However, due to point source pollution from industrial wastewater discharge from nearby construction sites and human settlements during the wet season, the average DIN and DIP content in the PRE was higher than in the tidal mangrove creeks, while nonpoint sources of pollution included agricultural runoff, domestic sewage, and shrimp farm. Cells poor in phosphorus easily absorb orthophosphate from waterbodies with lower amounts, making it a key source of nutrients for phytoplankton (Boney, 1986). Algae and other photosynthetic aquatic life, especially primary producers, are encouraged to thrive by phosphate compounds found in water. Indicating that these nutrients are removed from the mangrove region by the spring tide's inundation and tidal mixing, the average DIN and DIP concentrations were significantly higher at spring tide than at neap tide. The concentration of dissolved inorganic nitrogenous forms increased significantly during the wet season. In addition to anthropogenic sources, we may also blame these increases on more intensive breakdown of significant volumes of organic matter from mangrove trash and detritus (Pamplona et al., 2013). Salinity was crucial in helping particles release their phosphate content (Pamplona et al., 2013). The main indicator of an estuary's health is salinity, and salt content progressively rose to its peak during the winter dry season before sharply declining during the rainy season. Because of this, throughout the dry months, the salinity profiles along the creeks indicate a progressive decline in salinity landward from the creek mouth (Feb–June). On the other hand, during the monsoon and pre-monsoon seasons, a progressive rise in salinity has been noticed landward from the creek mouth (June–January). During high tide, water from the Bay of Bengal enters the estuary zone, increasing the salinity level. But at low tide, freshwater discharge from upstream rivers influences the study region's salinity, which is also supported by Sodre et al. (2011). Previous studies discovered similar variations in salinity with tide (Neeri, 1976; Mitra et al., 2011; Rahaman et al., 2013). In tropical estuarine environments, salinity is a major determinant of the geographical and temporal distribution of phytoplankton, zooplankton and frequently serves as an ecological barrier (Sodre et al., 2011; da Costa et al., 2011; da Costa et al., 2013). Salinity variations have a big impact on diatom proliferation. Salinity has also been identified as a significant factor contributing to changes in phytoplankton community structure in mangrove ecosystems (Hilaluddin et al., 2020). Diatoms require silica in addition to phosphate, ammonia, and nitrate in order to build their frustules and to encourage diatom growth (Krishnan et al., 2020). Silica is crucial in controlling how the phytoplankton community is composed (Sospedra et al., 2018). Due to the flushing of the surface mangrove sediments by tidal inundation and mixing during the dry season, the dissolved silica concentrations in the tidal mangrove creeks were greater than in the PRE. The PRE had greater dissolved silica concentrations than the tidal mangrove streams during the wet season. High rainfall and increased surface runoff during the wet season increased water turbidity and decreased transparency, which reduced light intensity and diatom density. Suspended sediments decreased light permeability and decreased diatom development (Hilaluddin et al., 2020). The main source of DSi entering the tidal estuary was freshwater runoff, with river runoff making up the majority of silica inputs during the rainy season (Krishnan et al., 2020). Ammonia makes up the majority of the DIN in mangrove sediments, with traces of nitrate and nitrite (Alongi et al., 1992). It is also known that the sediments are abundant in phosphate and silica. Greater levels of ammonia, silica, and phosphate in the water of the mangrove creeks in the Sundarbans are therefore anticipated to signify tidal flushing of nutrients from the mangrove zone.

The amount of flooding experienced by mangrove zones and tidal flats fluctuates over the course of a spring-neap cycles. Almost all of the water is retained within the main channel during a neap tide. However, the vegetated mangrove section is submerged during a spring tide (Tanaka and Choo, 2000; Cohen et al., 2004; Sodre et al., 2011). Key ecosystems for the transmission of nutrients from river drainage basins to nearby coastal areas are estuarine systems. These systems rank among the most prolific in the world, acting as spawning grounds for a variety of fish, crustacean, and mollusk species that are crucial for commerce (Sodre et al., 2011). Phytoplankton communities a kind of photoautotrophic organisms that use dissolved nutrients in the water column to make organic matter that is passed up the aquatic food web occur in these conditions (Sodre et al., 2011). Indicators of phytoplankton biomass and the trophic condition of estuarine and marine waters include chlorophyll-a concentrations (Steele, 1962); higher chlorophyll-a concentrations would result in higher production of phytoplankton biomass (Yao et al., 2010). Between spring and neap tides, there were notable variations in chlorophyll-a concentrations. Chlorophyll-a levels peaked at 6.85 μg/L at the spring tide in the tidal mangrove creeks. Chlorophyll-a levels at neap tides, however, were less than 4.89 μg/L, which is also supported by Tanaka and Choo (2000); Sodre et al., (2011); and Cavalcanti et al., (2018). The high phytoplankton biomass in the spring tide may represent the nutrients outwelling from the mangrove area by the inundation and tidal mixing of the spring tide. The nutrients outwelling from the mangrove region boosted the concentration of chlorophyll-a in the spring tide above the neap tide. However, because of the high nutrients that the mangroves supply to the creeks, which enable photosynthesis in the presence of light and thus encourage phytoplankton formation in both dry and wet seasons, chlorophyll-a concentrations were higher in tidal mangrove creeks than in the PRE. Based on chlorophyll-a concentrations, the Pasur River estuary (6.4 μg/L) and the tidal mangrove creeks (10.8 μg/L) imply "Mesotrophic" evaluation ratings, the estuarine zone may be characterized as "Fair."

Mangrove creeks have the potential to export massive amounts of organic matter and nutrients to the associated estuary and, eventually, to coastal marine habitats, which has a significant impact on the biological productivity of the estuary and coastal environment. Instead of being submerged at spring tide during the dry season, the Sundarbans mangrove swamp is submerged during the wet season. This causes salt and nutrients to outwell from the mangrove wetland via tidal mixing and conveyance.

## 5 Conclusion

According to the findings of the current study, multivariate statistical analysis showed that tide levels also had an impact on the quantities of dissolved inorganic nutrients and chlorophyll-a, concentrations in the study region. The results of this analysis showed that mangrove forests and related tidal mangrove creeks support significant chlorophyll-a production in estuary waters that is impacted by outwelling from mangrove forests and tidal mangrove creek sediments, in addition to nearshore production. Therefore, mangrove creeks played a crucial role as a supply of nutrients for PRE, consequently affecting its productivity. For the purpose of developing policy for mariculture activity in the PRE, the data supplied here will be used as a future reference, particularly by agencies responsible for fisheries management.

## 6 Limitations

The Pasur River estuary is classified as well mixed estuary (Shaha et al., 2022). As a result, the water samples were collected from a depth of 0–0.5 m (euphotic layer) instead of from the mid and bottom water.

## Declarations

### Author contribution statement

Jahid Hasan: Conceived and designed the experiments; Performed the experiments; Analyzed and interpreted the data; Wrote the paper.

Dinesh Chandra Shaha: Conceived and designed the experiments; Performed the experiments; Analyzed and interpreted the data; Contributed reagents, materials, analysis tools or data; Wrote the paper.

Sampa Rani kundu; Minhaz Ahmed: Conceived and designed the experiments; Wrote the paper.

Shahroz Mahean Haque; Farhana Haque: Analyzed and interpreted the data; Wrote the paper.

Md. Emranul Ahsan; Salman Ahmed: Performed the experiments; Wrote the paper.

Md. Iqbal Hossain; Mohammad Abdus Salam: Contributed reagents, materials, analysis tools or data; Analyzed and interpreted the data; Wrote the paper.

### Funding statement

Dr. Dinesh Chandra Shaha was supported by Research Management Wing [Area and project no. Marine Resources and PN 15], International Foundation for Science [W/5414-2].

### Data availability statement

Data will be made available on request.

### Declaration of interest’s statement

The authors declare no conflict of interest.

### Additional information

No additional information is available for this paper.
